# Editorial: Nutrition and oral health: at the micro-level

**DOI:** 10.3389/froh.2025.1772757

**Published:** 2026-01-30

**Authors:** Karlla Almeida Vieira, Diego Figueiredo Nóbrega, Lina Maria Marin

**Affiliations:** 1 Department of Dentistry, Cesmac University Center, Maceió, Alagoas, Brazil; 2 Faculty of Medicine - Federal University of Alagoas, Maceió, Alagoas, Brazil; 3 College of Dentistry, University of Saskatchewan, Saskatoon, SK, Canada

**Keywords:** chronic disease, nutrients, micronutrients, nutritional status, oral health, public health, odontogenesis

Research from around the world demonstrates that nutrition has a significant impact on oral health. Enamel strength, oral microbiota balance, and inflammation are all influenced by dietary patterns. While nutrients build tissue resilience, chronic diseases amplify damage through persistent inflammation and immune dysregulation. This editorial explores six recent studies to show how nutritional deficiencies, poor dietary habits, and systemic conditions drive oral disease progression — and why balanced nutrition remains essential for oral and overall health.

## The relationship between nutritional imbalance and tooth development and mineralization

Tooth development and enamel mineralization are highly susceptible to nutrient availability during pregnancy and early childhood. Developmental Defects of Enamel (DDE)—including fluorosis, Molar-Incisor Hypomineralization (MIH), and hypoplasia—are closely linked to deficiencies in calcium, phosphorus, protein, and vitamins A, D, and K (1). Dehailan and Martinez-Mier in their review indicated that Developmental Defects of Enamel (DDE), including fluorosis and MIH, arise from the interplay of nutritional, environmental, and biological variables that alter amelogenesis. Fluorosis is primarily attributed to elevated fluoride exposure from water, supplements, and early feeding, in conjunction with socioeconomic factors. MIH is more closely linked to vitamin D deficiency, perinatal issues, diseases, and antibiotic use, although the evidence on vitamin D is unclear due to methodological heterogeneity.

Fat-soluble vitamins (A, D, E, and K) are essential for many body functions, like vision, bone health, and immune system support. The body cannot produce these vitamins; they must be obtained through diet. A varied and balanced diet rich in vitamins is crucial for getting them, especially during periods of growth, pregnancy, breastfeeding, old age, or isolation. It is important to pay close attention to how they are absorbed and metabolized, as digestive problems, liver ailments, and chronic conditions can all lead to varying degrees of deficiency (2).

Khan et al. conducted a cross-sectional study in 2025 that explored NHANES 2011–2018 data from over 9,000 US youth aged 1–19 years, which revealed that increased dietary intake of phosphorus and fat-soluble vitamins (specifically A, E, and K) was associated with a decrease in dental caries across various age groups, with vitamin A being most protective for young children (1–5 years), vitamin E for older children (6–11 years), and vitamin K for adolescents (12–19 years). In this cross-sectional study, higher intake of vitamin C was linked to more caries in young children, but this association weakened when sugar intake was considered. While fruits and vegetables are optimal for vitamin C intake, fruit drinks, which are usually high in sugar, are a major source of vitamin C for people in the US. This indicates that sweet fruit beverages, rather than vitamin C itself, may have been responsible for the caries status. In contrast, greater phytate intake was linked to a lower caries incidence in young children, possibly due to its association with phosphorus-rich plant foods or other mechanisms, thereby contesting prior apprehensions about antinutrients.

## The relationship of macronutrient (carbohydrates, proteins, fats, and lipids) and micronutrient (vitamins and minerals) intake on oral health

Diet affects the oral microbiome and the body's response to inflammation throughout life, not just during development. High-sugar and high-fat/low-fiber diets promote pathogenic bacteria, lower plaque pH, and lead to caries and periodontitis (3). Fiber, antioxidants, and omega-3 fatty acids, on the other hand, help maintain a healthy microbial balance and lower inflammation (4).

Zhu et al. performed an NHANES-based cross-sectional study that found greater adherence to the Dietary Index for Gut Microbiota (DI-GM) was associated with a lower prevalence of periodontitis. Systemic inflammation biomarkers, particularly C-reactive protein and white blood cell count, elucidated this correlation, supporting a gut-oral axis in which microbiome-friendly diets (rich in fermented foods and polyphenols) may alleviate periodontal inflammation via anti-inflammatory metabolites, such as short-chain fatty acids. These results enhance prior studies on comprehensive food quality indices by highlighting DIGM's distinctive emphasis on the microbiome.

Casarin et al. linked dietary patterns and eating disorders to periodontal disease in their systematic review. Findings indicate that high-carbohydrate/sugar, high-fat, and low-fiber diets, as well as eating disorders like anorexia and bulimia, increase periodontal risk through nutritional deficiencies, microbiota disruption, and inflammation. Emerging evidence indicates that unhealthy dietary patterns— high in carbohydrates/sugars, fats, and low in fiber—as well as eating disorders, are associated with increased risk of periodontal diseases, exacerbated by nutritional and vitamin deficiencies. Balanced diets high in nutrients such as vitamins A, B, and C, calcium, zinc, and polyphenols seem to protect against these diseases. However, the roles of micronutrients like D, E, K, and magnesium are still not clear. Eating disorders are consistently associated with greater plaque accumulation and gingival inflammation.

## The relationship of chronic diseases on oral health

Systemic chronic illnesses worsen damage to oral tissues via pro-inflammatory cytokine networks and reactive oxygen species signaling. Eating disorders, on the other hand, cause cumulative damage through repeated acid exposure and micronutrient depletion.

Dietary habits may affect periodontal disease via many proposed processes. High intake of fermentable carbohydrates and deficiencies of vitamins C, D, and B-12 are risk factors for periodontitis. This is largely due to the effect these factors have on inflammation, oxidative stress, microbial balance, and metabolic health. Proinflammatory diets (e.g., high in refined sugars, saturated fats, processed foods, and low in fiber) promote periodontal disease by: increasing systemic low-grade inflammation via elevated pro-inflammatory cytokines, oxidative stress from Reactive Oxygen Species, and postprandial dysmetabolism; causing dysbiosis in the oral and gut microbiota by promoting harmful bacteria and lowering helpful ones; obesity, insulin resistance, and metabolic syndrome all make local periodontal inflammation worse via two-way mechanisms (hyperglycemia makes it easier for bacteria to grow) (5, 6).

Periodontitis, a prevalent chronic inflammatory disease affecting over 1 billion individuals worldwide, illustrates this interaction by generating bidirectional systemic sequelae, in which vitamin deficiencies often result in recurrent ulcers and prolonged wound healing in susceptible populations (7).

Usuga-Vacca et al. examined the correlation between consultations for oral disorders and systemic conditions. There were strong connections between oral and systemic consultations, driven by shared risk factors like poor biofilm control and high sugar consumption, with robust links to caries/gingivitis and moderate ones to malnutrition or certain circulatory issues. Socioeconomic indicators demonstrated a significant correlation with increased consultation rates for both oral and systemic disorders. Principal component analysis identified clusters linking caries/gingivitis to dietary pathologies and inequality indicators, circulatory diseases/diabetes to chronic periodontitis, and demographic factors to other disorders.

Rosa et al. showed emerging evidence that hypovitaminosis—specifically deficiencies in vitamins B12, folate, and C—constitutes a significant, modifiable factor in the aetiology of Recurrent Aphthous Stomatitis (RAS). The bidirectional oral-systemic axis is evident in mucosal disorders such as RAS, a common and distressing oral ulcerative condition with various aetiologies, including hypovitaminosis, a phenomenon gaining increasing recognition. Deficiencies undermine mucosal integrity, cellular repair, and immunological responses, often arising from malabsorption, nutritional inadequacy, or systemic disorders.

## Conclusion and recommendations

Developmental enamel defects, micronutrient protection against caries, vitamin deficiencies linked to oral ulcers, gut-oral microbial mediation, and systemic disease interactions all demonstrate that nutrition is a fundamental determinant of oral health. To optimize tooth development and combat oral diseases linked to systemic inflammation, nutrition and integrated care must be prioritized, especially in vulnerable periods and across vulnerable populations.

Prioritize Nutrient-Dense Diets for Lifelong Dental Health—During pregnancy, infancy, and childhood—when enamel is most vulnerable—emphasize foods rich in phosphorus and fat-soluble vitamins (A, E, and K) to support mineralization and reduce caries risk.
Adopt Integrated Public Health Policies—Oral and systemic disease management, tackling social determinants and shared risks like excess sugar, poor hygiene, and limited access. Target vulnerable groups to prevent caries, periodontitis, RAS, diabetes, hypertension, and obesity through coordinated, community-level interventions.
Enhance Screening and Multidisciplinary Collaboration—Screen patients with recurrent ulcers, periodontitis, or caries for hypovitaminosis and comorbidities using biochemical and social evaluations. Train dentists/hygienists to detect signs of eating disorders/malnutrition and integrate them into teams with nutritionists, physicians, psychiatrists, and psychologists for early referrals and holistic care.

Oral health at the micro-level is like nutritional status, which keeps the whole body healthy. By prioritizing nutrition across the life course, cycles of oral and chronic disease can be broken, thereby reducing global health disparities.
